# Occurrence of virulent and antibiotic-resistant Shiga toxin-producing *Escherichia coli* in some food products and human stool in Egypt

**DOI:** 10.14202/vetworld.2017.1233-1240

**Published:** 2017-10-15

**Authors:** Osman Mohamed Hamed, Maha Ahmed Sabry, Nawal A. Hassanain, Eman Hamza, Ahmed G. Hegazi, Marwa Badawy Salman

**Affiliations:** 1 Department of Zoonoses, Faculty of Veterinary Medicine, Cairo University, Cairo, Egypt; 2 Department of Zoonotic Diseases, Veterinary Research Division, National Research Centre, Giza, Egypt

**Keywords:** antibiotic resistance bacteria, cheese, DNA sequencing, human stool, meat, polymerase chain reaction, serotyping, shiga toxin-producing *Escherichia coli*

## Abstract

**Aim:**

Shiga toxin-producing *Escherichia coli* (STEC) represent a severe public health issue worldwide, causing life-threatening diseases in the human gastrointestinal tract. This study aimed to determine the occurrence of virulent and antibiotic-resistant STEC in retail meat and milk products and human stool samples and to characterize the genes encoding for virulence and antibiotic resistance among the identified STEC isolates.

**Materials and Methods:**

A total of 260 food samples were randomly collected from retail markets in different localities of El Giza Governorate, Egypt. 50 stool specimens were obtained from children that had diarrhea at Embaba Fever Hospital. All collected samples were initially subjected to bacteriological examination and serotyping, and then subsequently, the isolates were exposed to polymerase chain reaction application and sequencing for the identification of the virulence-related genes. Finally, the virulent STEC isolates were tested for antibiotic susceptibility.

**Results:**

Serotyping of the 76 biochemically identified isolates showed that 18 were STEC with a predominance of non-O157 (16) while 2 O157:K-serotype was detected only in one food and one human isolate. Molecular identification of the virulence genes illustrated that the minced meat showed the highest prevalence of STEC (8%) as compared to the other food products. In the humans, the O157 was the only serotype that expresses the Shiga toxin-associated gene (*eaeA*). Antibiotic susceptibility test displayed that 13 of the 17 food and human isolates (76.47%) were resistant to cephalothin (KF30). 9 of the 13 cephalothin-resistant isolates harbor the β lactamase (*bla_TEM_*)-resistant gene. All isolates were sensitive to chloramphenicol, ciprofloxacin, amikacin, and gentamicin. DNA sequencing and phylogenetic analysis of the *stx2-*positive minced meat isolate revealed a high genetic relatedness with beef minced meat from the USA and Australia.

**Conclusion:**

This study showed the predominance of non-O157 among the identified isolates. Minced meat showed the highest prevalence of STEC as compared to the other food products, and this work illustrates the necessity to consider the food products as a potential source of the non-O157 STEC serotypes. DNA sequencing and phylogenetic analysis revealed a high genetic relatedness with beef minced meat from the USA and Australia. This highlights the high probability of worldwide spread of such serotypes, signifying the importance of the one world concept.

## Introduction

*Escherichia coli* *(EC)* is normally found as normal flora in the intestinal tract of human and warm-blooded animals, but some strains have acquired pathogenic or toxigenic virulence factors that make them virulent for human and animals [1]. On the basis of somatic (O), flagellar (H), and capsular (K) antigens, diarrheagenic EC is divided into six pathotypes: Enteroaggregative, enterohemorrhagic/Shiga toxin-producing *E. coli* (STEC), enteroinvasive, enteropathogenic, enterotoxigenic, and diffuse adherent (*DAEC*) [2].

STEC represents a hazardous public health problem worldwide causing various human gastrointestinal tract diseases, including watery or bloody diarrhea, and might develop life-threatening diseases, such as hemorrhagic colitis, thrombotic thrombocytopenic purpura, (TTP) and hemolytic-uremic syndrome (HUS), and the latter is characterized by thrombocytopenia, microangiopathic hemolytic anemia, and acute renal failure [3]. The most important STEC serotypes which have been associated with human illness are O157, O111, O26, O103, O113, O91, O117, O118, O121, O145, O128, and O146 [4]. STEC infections are mainly foodborne infections; foods of high risk for transmission are meat products such as minced meat, sausage, hamburger and luncheon, and dairy products [5].

STEC strains are characterized by the production of two powerful phage-encoded cytotoxins causing tissue damage in humans and animals, called Shiga toxins or verotoxins (*stx1* and *stx2*) [6]. In addition, a number of accessory virulence factor genes such as the intimin (eae) and the enterohemolysin (hly) have been described [7].

Treatment of EC infection has been increasingly complicated worldwide by the emergence of resistance to most antibiotics. Among these, β lactams (e.g. penicillins, cephalosporins, cephamycins, and carbapenems), tetracycline, and aminoglycosides (streptomycin) are of particular interest [8]. The inappropriate use of antibiotics in animal production brought up resistance in commensal and pathogenic bacterial strains. It has been suggested that commensal EC might represent a reservoir of resistance genes for other bacteria [9]. One of the important mechanisms used by antibiotic-resistant EC is enzymes which hydrolyze β lactam antibiotics [10] such as extended spectrum β-lactamases (ESBLs). Many ESBLs are members of TEM (named after the patient temoneira) [11] which are capable to hydrolyze penicillins and first-generation cephalosporins [12].

Resistance to tetracycline is encoded by more than 40 genes (tet-genes), and they are divided into 11 classes, with a majority of classes (60%) encoding for membrane-associated efflux proteins. These efflux pumps selectively transport tetracycline from the cytosol to the periplasm, thereby limiting the access of tetracycline to the ribosomes in the cell [13]. *Tet (A)* is the most common efflux pump type found in commensal and clinical EC animal isolates [14]. The other resistance commonly detected in EC was against streptomycin and is mainly attributed to *aadA* type gene variants (*aadA1*, *aadA2*, and *aadA5*) [15,16]. Hence, the current study was aimed to determine the prevalence of STEC and to detect virulence genes (*stx1, stx2, eaeA*, and *hlyA*) as well as resistance determinants (*bla_TEM_*, *aadA2*, and *Tet A)* among the STEC isolates from examined retail meat and milk products and human fecal samples collected from fever/public hospital in Egypt.

## Materials and Methods

### Ethical approval

Ethical clearance to use human subjects was obtained from the designated health facility (National Research Centre, Giza, Egypt). Written consent was obtained from each person on information of the use of samples. This study was conducted in Giza Governorate, Egypt, at the period from January 2012 to August 2016.

### Collection of samples

A total of 200 meat samples including minced meat (n=50), luncheon (n=50), sausage (n=50), and beef burger (n=50), and 60 Karish cheese samples were collected from retail markets in randomly selected localities in El Giza Governorate. Fecal samples were obtained from 50 hospitalized children with diarrhea at Embaba Fever Hospital.

### Bacterial isolation

This method was performed according to the De Boer and Heuvelink [17]. Briefly, 25 g from each sample were transferred to tubes containing 225 ml of trypticase soya broth (TSB, Oxoid, England), blended, and incubated at 37°C for 24 h in an incubator (Sheldon MFG Inc., USA). A loopful from each of the previously incubated enrichment broth tubes was streaked over Eosin Methylene Blue agar (EMB, Oxoid, England) and Sorbitol McConkey agar plates (SMAC, Oxoid, England) and then incubated at 37°C for 24 h. Suspected colonies were stained with Gram stain and examined microscopically to detect Gram-negative rods. The presumptive colonies were purified on agar slopes and were incubated at 37°C for 24 h for further identification.

### Biochemical identification

Presumptive colonies were confirmed biochemically using GNB 12 A kit (Oxoid, England) for Gram-negative bacilli.

### Serotyping

The identified EC isolates were serotyped by slide agglutination test in the central laboratories of Ministry of Health and Population (Cairo, Egypt) using standard polyvalent and monovalent EC antisera according to Edwards and Ewing [18].

### Antibiotic sensitivity test

Confirmed isolates tested using disk diffusion method on Muller-Hinton agar plates (Oxoid) for susceptibility to commonly used antibiotics (Oxoid, UK), amikacin (AK30, 30 mcg), amoxicillin/clavulanic acid (AMC30, 20/10 mcg), ampicillin (AMP 10, 10 mcg), cefotaxime (CTX30, 30 mcg), ceftriaxone (CR30, 30 mcg), cephalothin (KF30, 30 mcg), chloramphenicol (C30, 30 mcg), ciprofloxacin (CIP5, 5 mcg), gentamicin (GN10, 10 mcg), streptomycin (S10, 10 mcg), and tetracycline (TE30, 30 mcg). The results were interpreted according to CLSI [19].

### Molecular identification of STEC isolates

#### DNA extraction

Genomic DNA was extracted from pure EC colonies using QIAamp DNA Mini kit (Qiagen, Germany) according to the manufacturer’s recommendations.

#### Polymerase chain reaction (PCR) amplification

Primers for the studied virulence and antibiotic resistance genes (Midland Certified Reagent Company Oligos, USA). The PCR mixture for a total reaction volume of 25 μl consisted of 6 μl DNA template from each isolate, 12.5 μl of Emerald PCR Master Mix (Takara, Japan), 1 μl of 20 pmol of each primer, and 4.5 μl of PCR grade water. The reactions were performed in T3 thermal cycler (Biometra, Germany) under different conditions for each primer set according to the previous studies as stated in Table-1 [20-26]. The PCR products (15μl each) were electrophoresed on 1.5% agarose gel (Applichem, Germany, GmbH). A 100 bp DNA Ladder (Qiagen, Germany) was used to determine the size of the product. The gel was photographed using gel documentation system (Alpha Innotech, Biometra, Germany), and the data were analyzed using computer software.

**Table-1:** Primers used for virulence- and antibiotic resistance-related genes of the STEC isolates.

Gene	Primers sequence	Amplified segment (bp)	References
*stx1*	F: ATG TCA GAG GGA TAG ATC CA	185 bp	[20]
	R: TAT AGC TAC TGT CAC CAG ACA AT		
stx1*	F: GGTGACTCTAGTAGGTCACA	614 bp	[21]
	R: GTATTACCTCCCCCTAAGTC		
*stx2*	F: CCATGACAACGGACAGCAGTT	779 bp	[21]
	R: CCTGTCAACTGAGCAGCACTTTG		
*eae A*	F: ATG CTT AGT GCT GGT TTA GG	248 bp	[22]
	R: GCC TTC ATC ATT TCG CTT TC		
*hlyA*	F: AACAAGGATAAGCACTGTTCTGGCT	1177 bp	[23]
	R: ACCATATAAGCGGTCATTCCCGTCA		
*blaTEM*	F: ATCAGCAATAAACCAGC	516 bp	[24]
	R: CCCCGAAGAACGTTTTC		
*aadA2*	F: TGTTGGTTACTGTGGCCGTA	622 bp	[25]
	R: GATCTCGCCTTTCACAAAGC		
*Tet (A)*	F: GGTTCACTCGAACGACGTCA	576 bp	[26]
	R: CTGTCCGACAAGTTGCATGA		

Primers used for amplification of stx1 gene included in sequencing

#### Sequence analysis

The amplicons of *stx1* and *stx2* were purified using QIA quick PCR Product extraction kit. (Qiagen Inc., Valencia CA) and then sequenced in Macrogen Company (Korea) using Applied Biosystems 3130 automated DNA Sequencer (USA). The nucleotide sequences were analyzed using BioEdit 7.0.4.1 program. The obtained nucleotide sequences were compared with those available in public domains using NCBI-BLAST server and were deposited in the GenBank Database. Sequence alignments and phylogenetic comparisons of the sequences for the examined genes were performed using MegAlign module of Lasergene DNAStar software.

## Results

**Figure-1:**
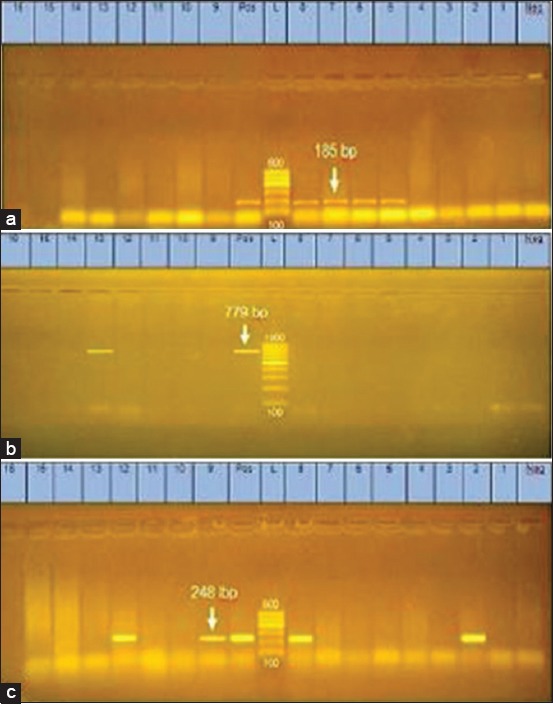
Agarose gel electrophoresis of polymerase chain reaction products amplified for virulence genes: (a) *stx1* gene. Lanes (L): DNA ladder (100 bp). Lane (Pos): Positive control. Lane (Neg): Negative control. Lanes 5-8 display positive Shiga toxin-producing *Escherichia coli* (STEC) isolates that showed specific bands at 185 bp. Lanes 1-4 and 9-16 are isolates negative to *stx1* gene. (b) *Stx2* gene Lane (L): DNA ladder (100 bp). Lane (Pos): Positive control. Lane (Neg): Negative control. Lane 13: Positive STEC isolates showing specific bands at 779 bp. Lanes 1-8, 9-12 and 14-16: Negative isolates for *stx2* gene. (c) *eae A* gene. Lane (L): DNA ladder (100 bp). Lane (Pos): Positive control. Lane (Neg): Negative control. Lanes 2, 8, 9, and 12: Positive STEC isolates showing specific bands at 248 bp. Lanes 1, 3-7, 10, 11, and 13-16: Isolates negative to *eaeA* gene.
- Antibiotic-resistant non-O157 serotypes were predominant in stools from diarrheic children (Table-3, Figure-2a and b).

**Figure-2:**
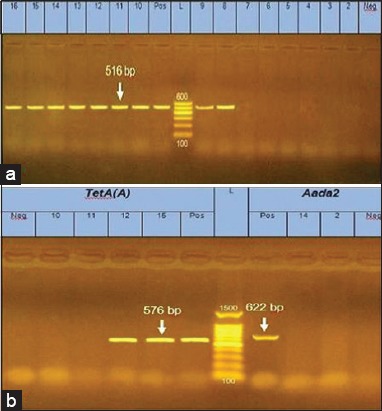
Agarose gel electrophoresis of polymerase chain reaction products for determinants of antibiotic resistance. (a) *bla TEM* gene in β-lactams resistant isolates. Lanes (L): DNA ladder (100 bp). Lane (Pos): Positive control. Lane (Neg): Negative control. Lanes 8-16: Positive STEC isolates showing specific bands at (516 bp). Lanes 2-7 isolates negative to *bla TEM* gene. (b) *Tet (A)* and *Aada2* genes in tetracycline and streptomycin-resistant isolates. Lane (L): DNA ladder (100 bp). Lane 1 (Neg): Negative control for *Aada2* gene. Lanes (2 and 14): negative isolates for *Aada2* gene. Lane 4 (Pos): Positive control for *Aada2* gene. Lane 6 (Pos): Positive control for *Tet (A)* gene. Lanes (12 and 15): Positive isolates for *Tet (A)* gene showing specific bands at (576 bp) Lane s (10 and 11): Negative isolates for *Tet (A)* gene. Lane 11 (Neg): Negative control for *Tet (A)* gene.
- Phylogenetic analysis of *stx2*-expressing STEC minced meat isolate.

- The occurrence of virulent β-lactam and tetracycline-resistant non-O157 STEC serotypes among meat products and Karish cheese is shown in Table-2, Figures-1 and 2.

**Table-2:** Prevalence of Shiga toxins and intimin among O157 and non-O157 serotypes in food products and their antibiotic resistance profiles.

Type of samples (total no.)	Serotypes	Virulence genes			Antibiotic resistance profile	Resistance genes	
	
		*stx1*	*stx2*	*eaeA*		*blaTEM*	*tetA*
Minced meat (50)	O125:K70	-	−	−	KF30	−	nt
	O125:K70	+	−	−	KF30	+	nt
	O26:K60	+	−	+	KF30	−	nt
	O103:K−	−	+	−	KF30, TE30, AMP10	+	−
Luncheon-50	O26:K60	−	−	−	KF30	+	nt
	O111:K58	−	−	−	Sensitive to all	nt	nt
Beef burger (50)	O125:K70	−	−	+	CTX30	nt	nt
Sausage-50	O125:K70	−	−	−	CTX30	nt	nt
	O124:K72	+	−	−	KF30	−	nt
	O157:K−	−	−	−	KF30	+	nt
	O111:K58	−	−	−	KF30, TE30	+	−
	O145:K−	−	−	−	TE30, AMP10	nt	+
Karish cheese (60)	O124:72	−	−	−	CTX30	nt	nt
	O124:72	+	−	+	KF30	+	nt

nt=Not tested, +=Positive, −=Negative

**Table-3:** Occurrence of Shiga toxins and intimin among O157 and non-O157 serotypes and their antibiotic resistance in diarrheic children stool specimens.

Total number	Serotypes	Virulence genes			Antibiotic resistance profile	Resistance genes	
	
		*eaeA*	*stx2*	*stx1*		*blaTEM*	*TetA*
50	O55:K59	−	−	−	KF30, TE30	+	+
	O55:K59	−	−	−	KF30, S10	−	nt
	O111:K58	−	−	−	KF30	+	nt
	O157:K−	−	−	+	AMC30, CR30, F30, S10	+	nt

nt=Not tested, +=Positive, −=Negative

**Figure-3:**
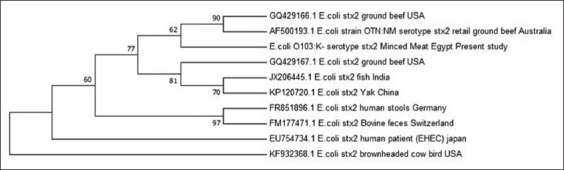
Phylogenetic analysis of Shiga toxin 2-producing *Escherichia coli* strain isolated from minced meat (KY884001).

The STEC isolates were sequenced for *stx2* gene, and a phylogenetic tree was constructed based on alignment with nine *stx2* genes retrieved from NCBI database that showed high homology with our sequence. The phylogenetic analysis demonstrates that the sequence of the minced meat isolates obtained in the present study (KY884001) was clustered with other two beef minced meat *E. coli* isolated from Australia (AF500193) and USA (GQ429166.1), indicating that our isolate might be the source of these two isolates. Interestingly, our isolate shared a common ancestor with two isolates from Asia (fish isolate from India, JX206445; yak isolate from China, KP120720.1) and two isolates from Europe (bovine fecal isolate from Switzerland, FM177471.1; human fecal isolate from Germany, FR851896.1) (Figure-3).

## Discussion

STEC is recognized as one of the most important recently emerged group of foodborne pathogens responsible for serious outbreaks worldwide [27,28]. In this study, the overall prevalence of STEC in the examined meat products was 3% (6 out of 200) which is higher than 0.3 recorded by Loukiadis *et al*. [29] from France and similar to Hessain *et al*. [30] in Saudi Arabia. Other studies reported higher prevalence in meat products that range from 9.38% in Egypt [31] to 11.6% in Romania [32]. These variations in the prevalence rate of STEC might be explained by the type of samples examined their source as well as a method of detection. In this regard, in this study, the highest percentage of STEC was found in minced meat (8%, 4 out of 50) as compared to beef burger and sausage that showed the presence of one STEC isolate. In contrast, the luncheon isolates were STEC negative. Raw meat was shown to contain high prevalence of STEC [33,34] which might explain their presence in sausage and beef burger since these products are prepared from raw meat and not cooked but preserved by lactic acid fermentation.

One of the most popular Egyptian diets is cheese due to its high protein content, low fat, and price [35]. Cheese, especially those made from raw milk like Karish, has been incriminated in recent foodborne outbreaks [36]. Our results showed contamination of Karish cheese samples with STEC in a percentage of 1.7% (1 out of 60) that is similar to other studies performed in Egypt [37].

Like other studies performed in Egypt [31,38], the non-O157 serotypes (O125, O26, O111, O145, and O103) were most commonly present in food products. The O157 serotype was isolated only from sausage but not from the other food products. This finding is similar to other reports [33,39] that demonstrated the inability to isolate the O157 serotype from minced meat, burger, and luncheon.

Molecular identification of the virulence genes confirmed the previous report [40] that *stx1* predominates over *stx2* in STEC isolates from food products. In this study, the s*tx1, stx2*, and *eaeA* genes were harbored mainly by minced meat STEC isolates. This is consistent with an Indian study performed by Sethulekshmi *et al*. [34] and in contrast with Dambrosio *et al*. [41] who stated that none of the meat STEC isolates harbored *stx1* or *stx2* genes. Interestingly, one of the two Karish STEC isolates carried both *stx1* and *eaeA* genes. However, the intimin gene (*eaeA*) acts as an accessory factor that is thought to enhance the virulence of STEC, and some STEC strains not harboring *eaeA* have been shown to cause human illnesses [42]. None of our STEC isolates carries *hlyA* gene, and this agrees with Abd El-Tawab *et al*. [43] and Khatib *et al*. [44]. This might be due to the presence of *iutA* gene encoding aerobactin which compensates for the absence of *hlyA* gene [45]. Further study is needed to examine whether our isolates carry *iutA* gene instead of *hlyA* because the isolates usually have either *iutA* or *hly*, but rarely both genes [45].

The public health importance of EC was examined in diarrheic children. Like in meat products, the non-O157 was the common serotype isolated from the diarrheic children in the present study. Although the O157 is the most frequent serotype causing human illnesses, there is rising evidence that non-O157 serotypes are linked to human outbreaks [46,47]. Our results provide additional evidence for the possible association of non-O157 serotypes with clinical disease in humans. Furthermore, the human isolates expressed neither *stx1* nor *stx2* genes except for one isolate of the O157 serotype were found to carry *eaeA* gene.

Examination of antibiotic susceptibility patterns of the animal and human isolates revealed that 17 of the 18 serotyped STEC isolates exhibited resistance to one or more antibiotic agents, and the majority of them (13 of the 17 [76.47%]) were resistant to cephalothin (KF30) irrespective to their origin (food or humans). Among the 13 KF30-resistant STEC isolates, nine harbor the *blaTEM*-resistant genes which explain the resistance to cephalothin as it is a β-lactam first-generation cephalosporin which gets hydrolyzed by the TEM β-lactamase [48]. Interestingly, two of the KF30-resistant strains were also tetracycline resistant with one of them carry *tetA* resistance gene. Resistance to cephalothin and tetracycline in EC strains isolated from different sources is consistent with other studies performed on different type of samples worldwide [49-53]. This highlights the widespread and lengthy use of both antibiotics for the treatment of human diseases and as a growth promoter in animals. Interestingly, all isolates were susceptible to chloramphenicol, ciprofloxacin, amikacin, and gentamicin, signifying the effective use of such antibiotics in the control of infection with STEC in animals and humans. This agrees with the study of Jiao *et al*. **[**54] and Jeyasanta *et al*. [55] who reported that amikacin and ciprofloxacin are among the effective antibiotics used to treat EC infection.

Strikingly, the *Aada2* resistance gene was not detected in any of the streptomycin-resistant isolates, suggesting that such resistance might be mediated by other yet undescribed genes [56]. The present results of antibiotic susceptibility agree and disagree with other studies, pointing to the variability in antibiotic resistance pattern according to the type of isolates, time, and development of multiple drug-resistant EC [57].

Phylogenetic analysis of the *stx2* gene from our minced meat STEC isolate showed a high genetic relatedness with *stx2* genes from beef minced meat STEC isolates from Australia and the USA. This might be due to the fact that the minced meat samples included in the present study are imported product. Moreover, the current *stx2* gene shared a common origin with those from different types of samples such as Indian fish, Chinese yak, Swiss bovine feces, and German human stools. Our results highlight the possible transmission of *E.C*carrying such gene worldwide through trade.

## Conclusion

In this study, minced meat showed the highest prevalence of STEC as compared to the other food products. Antibiotic susceptibility test displayed that majority of the food, and human isolates (76.47%) were resistant to cephalothin. DNA sequencing and phylogenetic analysis of the *stx2-*positive minced meat isolate revealed a high genetic relatedness with beef minced meat from the USA and Australia. This highlights the high probability of worldwide spread of such serotypes, signifying the importance of the one world concept.

### Significance statements

This study focused on the public health importance of the meat and meat products (minced meat and sausage) as a source of STEC, especially non-O157:H7 and causing human infections and outbreaks. In this study, all STECs were 100% susceptible to chloramphenicol, ciprofloxacin, amikacin, and gentamicin, signifying that these antibiotics could be of choice to be used in control of infectious diseases of STEC in human. On the other hand, the presence of antimicrobial-resistant EC may represent a reservoir of resistance genes for other bacteria such as beta-lactamase *blaTEM*, *TetA*, and *aadA2* and therefore decrease the efficacy of treatment of other bacterial infections. DNA sequencing and phylogenetic analysis of *stx2* gene from our minced meat STEC isolate with available sequences in NCBI revealed that these genes are closely related to that isolated from different countries, especially Australia and USA, which increase the role of importation of meat and meat products in the transmission of EC carrying such gene worldwide.

## Authors’ Contributions

OMH: Developed the concept and write the manuscript, MAS: Developed the concept and apply PCR assay, NAH: Developed the concept, apply bacteriological examination, and write the manuscript, EH: Developed the concept, share in bacteriological examination and write the manuscript, AGH: Developed the concept and share in bacteriological examination, and MBS (Corresponding author): Developed the concept, prepare samples, apply bacteriological examination, and apply PCR assay. All authors read and approved the final manuscript.
